# Supplementary material for: Electron cryo-microscopy of bacteriophage PR772 reveals the elusive vertex complex and the capsid architecture
Source: eLife. 2019 Sep 12;8:e48496. doi: 10.7554/eLife.48496 (PMC6750898; doi:10.7554/eLife.48496)
Supplement: Supplementary file 1. [file elife-48496-supp1.docx]

Sequence-guided structure alignment of most proteins from PR772 and PRD1 yielded poor RMSD values even though they have high sequence identity. So, secondary-structure based alignment (without the use of sequence alignment) was also performed. We see a significant variation in the models for P31, P30, P16 and minor variations in the overall P3 structure. The P31 protein from PR772 has 100% protein sequence identity with P31 from PRD1, but the model comparison reveals a RMSD of 16.7Å with significant registry error in the model from PRD1. P30 has a 97.6% sequence identity and the model comparation reveals a RMSD of 4 Å. P16 with a sequence identity of 96%, has a RMSD of 5.49 Å. P3 has a 99.7% protein sequence identity and the model comparison shows an overall RMSD of 1Å but the most crucial and functionally important C-terminal and N-terminal regions show a RMSD of 2.3Å and 3.0Å respectively.

Length of proteins and their traced part in the current atomic model of the asymmetric unit of PR772 is shown below.

| Protein | Chain | Length | Model Trace | Regions |
| --- | --- | --- | --- | --- |
| P3 | A | 395 | 390 | 3-392 |
|  | B | 395 | 377 | 14-390 |
|  | C | 395 | 394 | 2-395 |
|  | D | 395 | 380 | 12-391 |
|  | E | 395 | 379 | 12-390 |
|  | F | 395 | 394 | 2-395 |
|  | G | 395 | 388 | 6-393 |
|  | H | 395 | 374 | 11-384 |
|  | I | 395 | 394 | 2-395 |
|  | J | 395 | 390 | 3-392 |
|  | K | 395 | 377 | 14-390 |
|  | L | 395 | 392 | 4-395 |
| P30 | M | 84 | 84 | 1-84 |
| P16 | N | 117 | 51 | 30-58, 96-117 |
| P5 | O | 340 | 124 | 1-124 |
|  | P | 340 | 124 | 1-124 |
|  | Q | 340 | 124 | 1-124 |
| P31 | R | 126 | 125 | 2-126 |
|  | S | 126 | 125 | 2-126 |

**P31**

Protein Sequence Identity: 100%

**Sequence-Guided Structure Alignment**

**Chimera** [1]


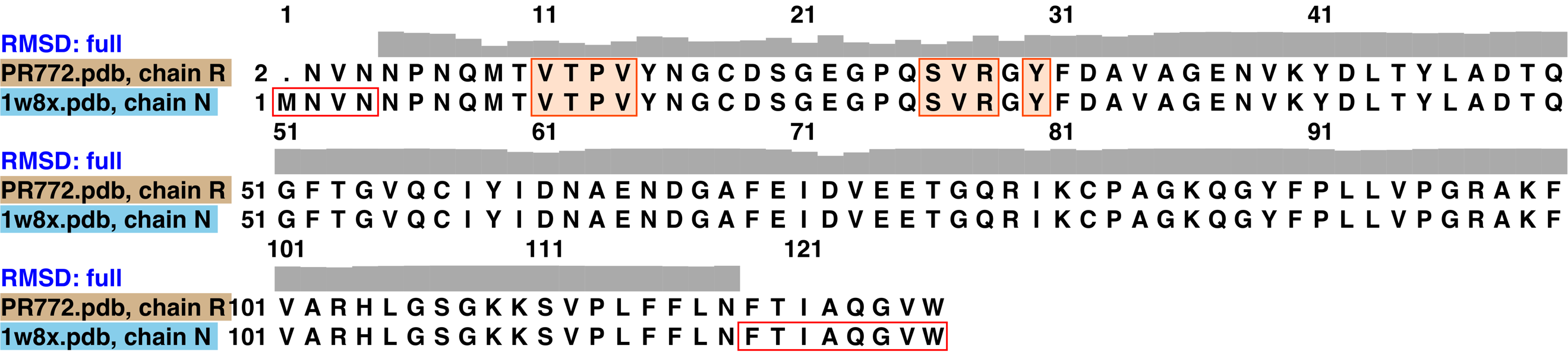


Overall RMSD: 16.68 Å

**SuperPose** [2]

| Local RMSD   \|  \| \| --- \| \| \|  \| Alpha Carbons \| Back Bone \| Heavy \| All \| \| --- \| --- \| --- \| --- \| --- \| \| RMSD \| 3.08 \| 3.08 \| 5.26 \| 5.26 \| \|  \|  \|  \|  \|  \| \| Atoms \| 13 \| 52 \| 97 \| 97 \| \|  \|  \|  \|  \|  \| \| \| \| Structure \| Residues \| \| --- \| --- \| \| PDBA chain 'R' \| 21-33 \| \| 1W8X chain 'N' \| 21-33 \| \| |
| --- | --- | --- | --- | --- | --- | --- | --- | --- | --- | --- | --- | --- | --- | --- | --- | --- | --- | --- | --- | --- | --- | --- | --- | --- | --- | --- | --- | --- | --- | --- | --- | --- | --- | --- |
| Global RMSD   \|  \| \| --- \| \| \|  \| Alpha Carbons \| Back Bone \| Heavy \| All \| \| --- \| --- \| --- \| --- \| --- \| \| RMSD \| 17.13 \| 17.06 \| 17.48 \| 17.48 \| \|  \|  \|  \|  \|  \| \| Atoms \| 113 \| 452 \| 864 \| 864 \| \|  \|  \|  \|  \|  \| \| \| \| Structure \| Residues \| \| --- \| --- \| \| PDBA chain 'R' \| 6-118 \| \| 1W8X chain 'N' \| 6-118 \| \| |

**Secondary Structure Based Alignment**

**Chimera**


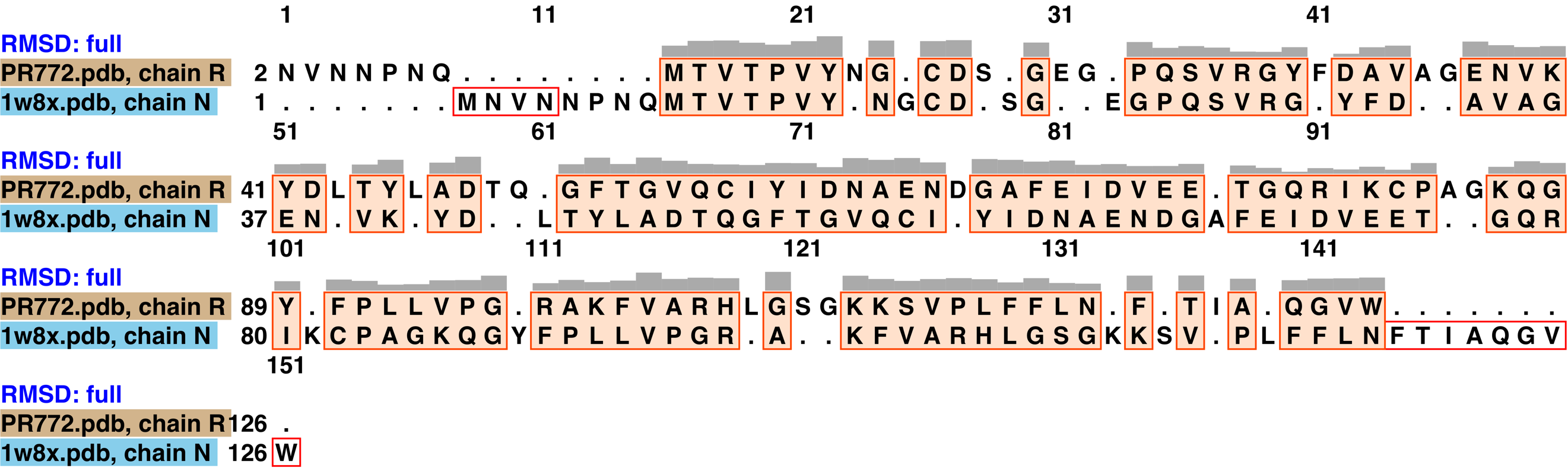


Structural Distance Measure (cutoff 5.0): 46.735

Q-score: 0.427

**P30**

Protein Sequence Identity: 97.6%

**Sequence-Guided Structure Alignment**

**Chimera**


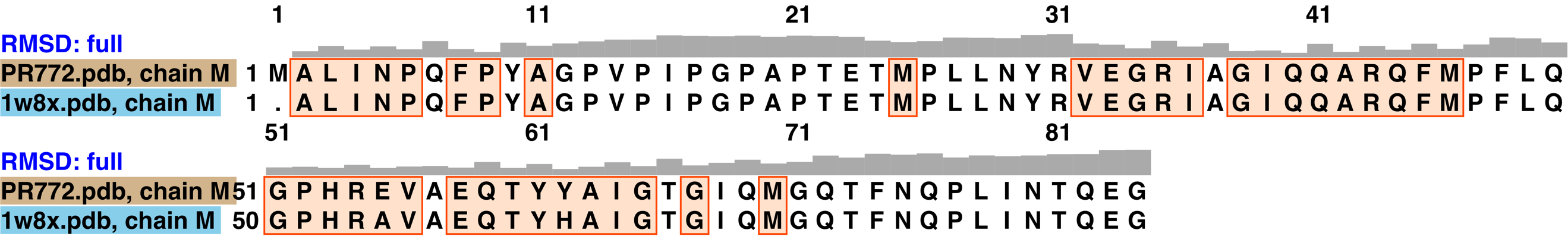


Overall RMSD: 3.97 Å

**SuperPose**

| Local RMSD   \|  \| \| --- \| \| \|  \| Alpha Carbons \| Back Bone \| Heavy \| All \| \| --- \| --- \| --- \| --- \| --- \| \| RMSD \| 3.53 \| 3.50 \| 4.91 \| 4.91 \| \|  \|  \|  \|  \|  \| \| Atoms \| 83 \| 332 \| 636 \| 636 \| \|  \|  \|  \|  \|  \| \| \| \| Structure \| Residues \| \| --- \| --- \| \| PDBA chain 'M' \| 2-84 \| \| 1W8X chain 'M' \| 1-83 \| \| |
| --- | --- | --- | --- | --- | --- | --- | --- | --- | --- | --- | --- | --- | --- | --- | --- | --- | --- | --- | --- | --- | --- | --- | --- | --- | --- | --- | --- | --- | --- | --- | --- | --- | --- | --- |
| Global RMSD   \|  \| \| --- \| \| \|  \| Alpha Carbons \| Back Bone \| Heavy \| All \| \| --- \| --- \| --- \| --- \| --- \| \| RMSD \| 3.53 \| 3.50 \| 4.91 \| 4.91 \| \|  \|  \|  \|  \|  \| \| Atoms \| 83 \| 332 \| 636 \| 636 \| \|  \|  \|  \|  \|  \| \| \| \| Structure \| Residues \| \| --- \| --- \| \| PDBA chain 'M' \| 2-84 \| \| 1W8X chain 'M' \| 1-83 \| \| |

**Secondary Structure Based Alignment**

**Chimera**


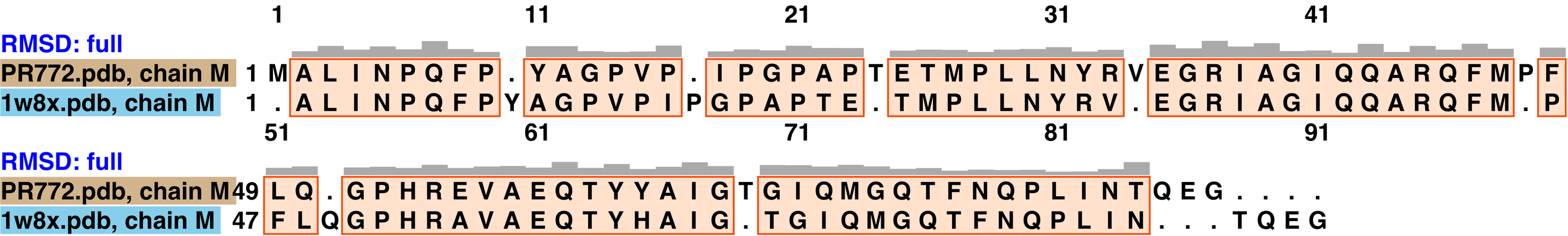


Structural Distance Measure (cutoff 5.0): 29.122

Q-score: 0.670

**P16**

Protein Sequence Identity: 94%

**Sequence-Guided Structure Alignment**

**Chimera**


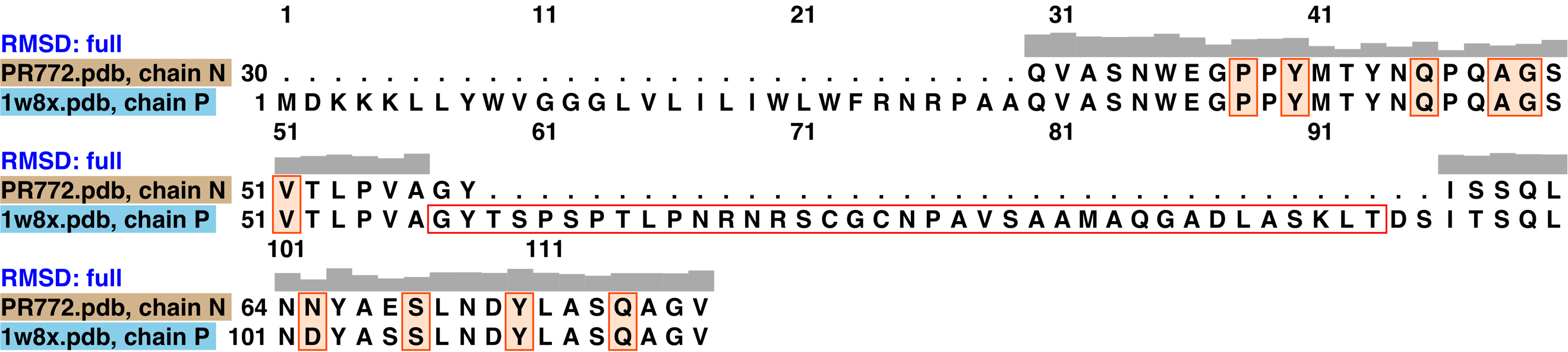


Overall RMSD: 5.49 Å

**SuperPose**

| Local RMSD   \|  \| \| --- \| \| \|  \| Alpha Carbons \| Back Bone \| Heavy \| All \| \| --- \| --- \| --- \| --- \| --- \| \| RMSD \| 4.39 \| 4.20 \| 5.82 \| 5.82 \| \|  \|  \|  \|  \|  \| \| Atoms \| 50 \| 201 \| 366 \| 366 \| \|  \|  \|  \|  \|  \| \| \| \| Structure \| Residues \| \| --- \| --- \| \| PDBA chain 'N' \| 30-56, 57-58, 96-117 \| \| 1W8X chain 'P' \| 30-56, 94-95, 96-117 \| \| |
| --- | --- | --- | --- | --- | --- | --- | --- | --- | --- | --- | --- | --- | --- | --- | --- | --- | --- | --- | --- | --- | --- | --- | --- | --- | --- | --- | --- | --- | --- | --- | --- | --- | --- | --- |
| Global RMSD   \|  \| \| --- \| \| \|  \| Alpha Carbons \| Back Bone \| Heavy \| All \| \| --- \| --- \| --- \| --- \| --- \| \| RMSD \| 4.39 \| 4.20 \| 5.82 \| 5.82 \| \|  \|  \|  \|  \|  \| \| Atoms \| 50 \| 201 \| 366 \| 366 \| \|  \|  \|  \|  \|  \| \| \| \| Structure \| Residues \| \| --- \| --- \| \| PDBA chain 'N' \| 30-56, 57-58, 96-117 \| \| 1W8X chain 'P' \| 30-56, 94-95, 96-117 \| \| |

**Secondary Structure Based Alignment**

**Chimera**


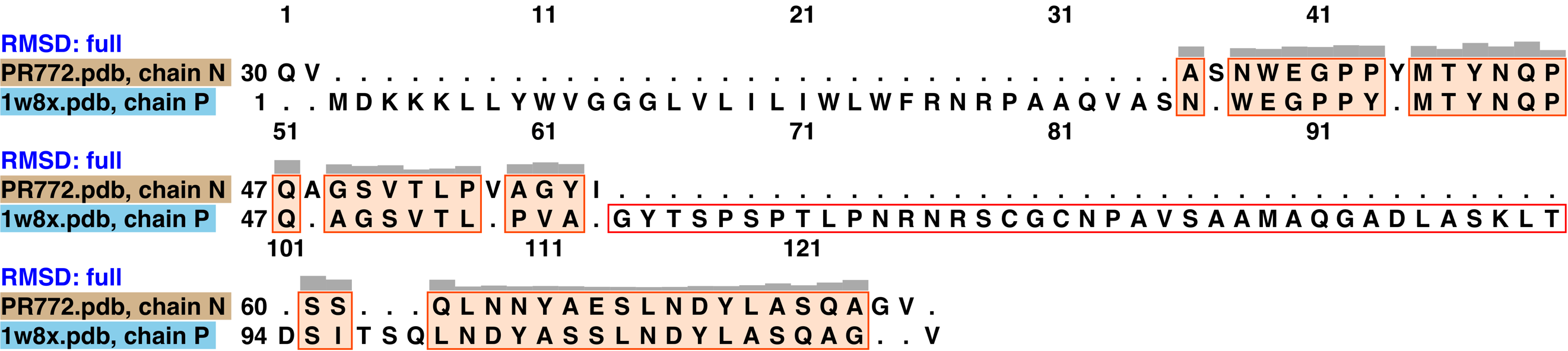


Structural Distance Measure (cutoff 5.0): 32.599

Q-score: 0.345

**P3 (Typical Case)**

Protein Sequence Identity: 99.7%

**Sequence-Guided Structure Alignment**

**Chimera**


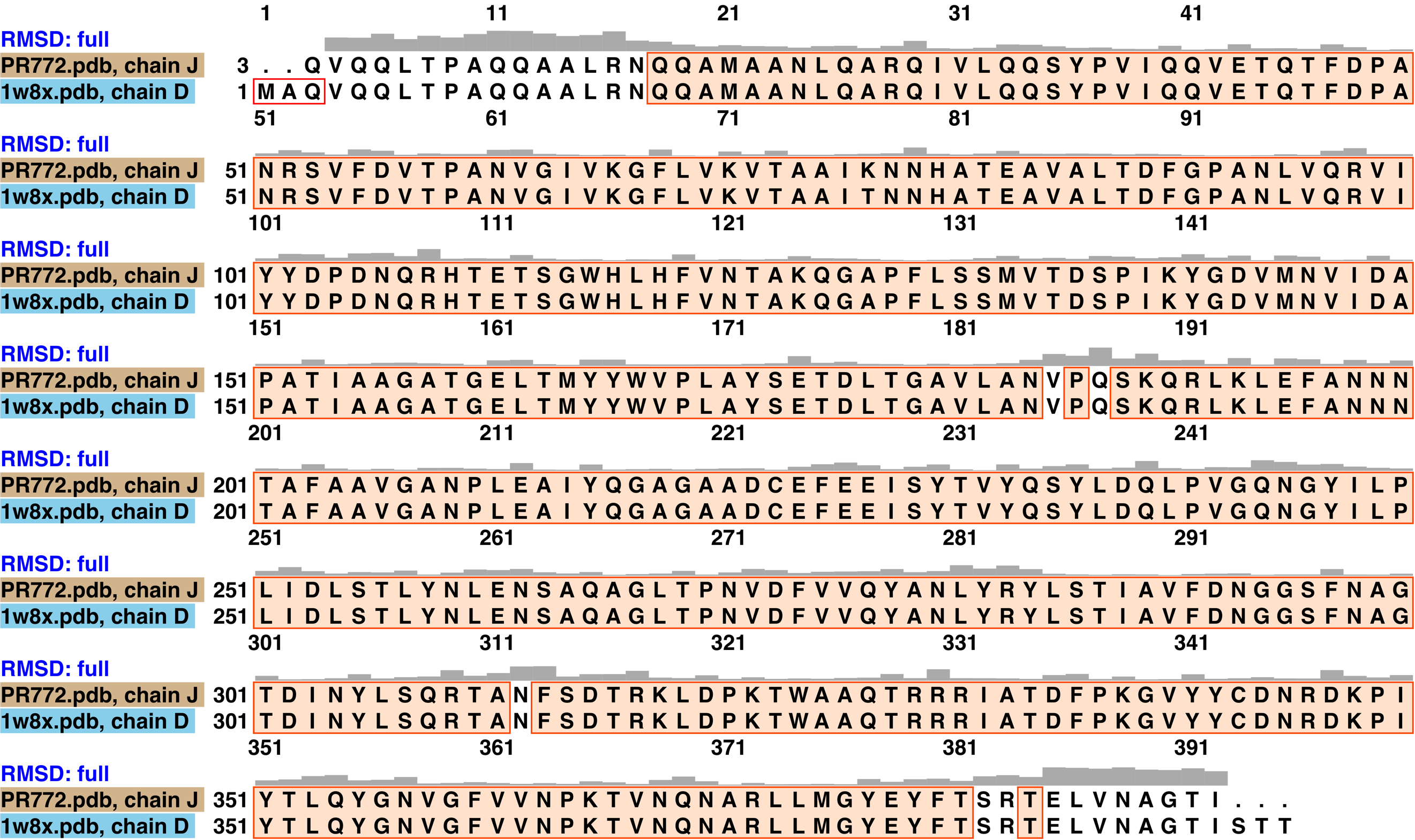


Overall RMSD: 1.18 Å

RMSD first 20 C-terminal residues (1-20): 2.26 Å

RMSD last 20 N-terminal residues (376 - 395): 3.04 Å

**SuperPose**

| Local RMSD   \|  \| \| --- \| \| \|  \| Alpha Carbons \| Back Bone \| Heavy \| All \| \| --- \| --- \| --- \| --- \| --- \| \| RMSD \| 1.17 \| 1.13 \| 1.83 \| 1.83 \| \|  \|  \|  \|  \|  \| \| Atoms \| 389 \| 1556 \| 3023 \| 3023 \| \|  \|  \|  \|  \|  \| \| \| \| Structure \| Residues \| \| --- \| --- \| \| PDBA chain 'J' \| 4-392 \| \| 1W8X chain 'D' \| 4-392 \| \| |
| --- | --- | --- | --- | --- | --- | --- | --- | --- | --- | --- | --- | --- | --- | --- | --- | --- | --- | --- | --- | --- | --- | --- | --- | --- | --- | --- | --- | --- | --- | --- | --- | --- | --- | --- |
| Global RMSD   \|  \| \| --- \| \| \|  \| Alpha Carbons \| Back Bone \| Heavy \| All \| \| --- \| --- \| --- \| --- \| --- \| \| RMSD \| 1.17 \| 1.13 \| 1.83 \| 1.83 \| \|  \|  \|  \|  \|  \| \| Atoms \| 389 \| 1556 \| 3023 \| 3023 \| \|  \|  \|  \|  \|  \| \| \| \| Structure \| Residues \| \| --- \| --- \| \| PDBA chain 'J' \| 4-392 \| \| 1W8X chain 'D' \| 4-392 \| \| |

SuperPose difference distance map for P3


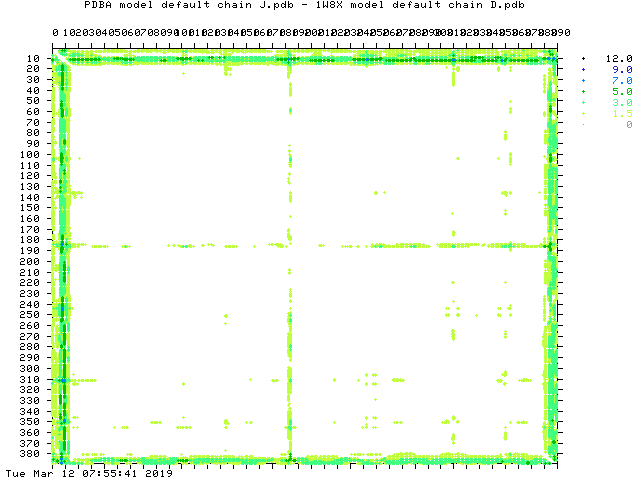


**Secondary Structure Based Alignment**

**Chimera**


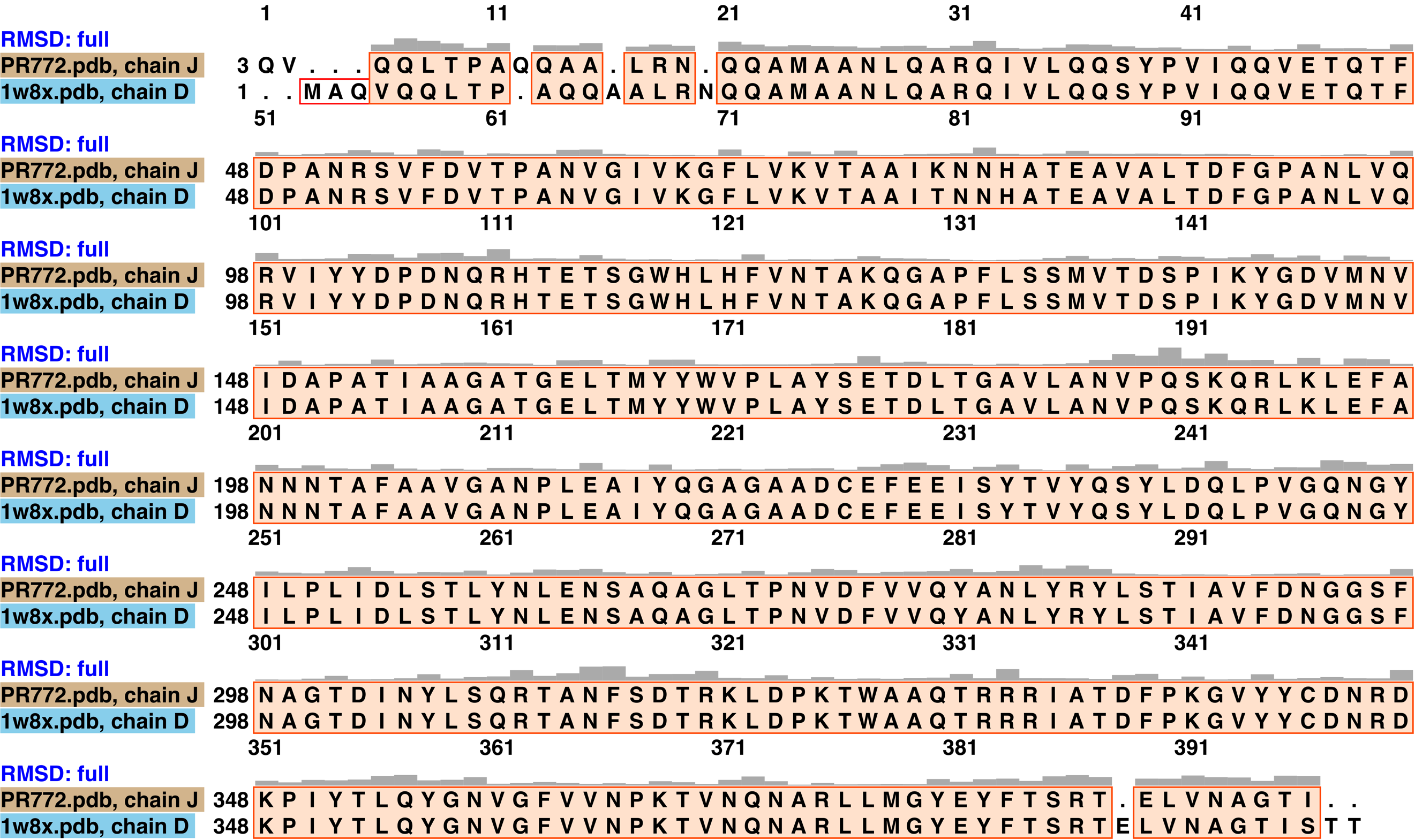


Structural Distance Measure (cutoff 5.0): 14.121

Q-score: 0.928

**Note:**

- RMSD: Root Mean Square Deviation.
- Structural Distance Measure [3] : It is zero for identical structures and increases as the similarity decreases.
- Q-score [4] : Values range from zero for dissimilar or poorly superimposed secondary structures to 1 for identical secondary structures.

**Reference**

1. Meng EC, Pettersen EF, Couch GS, Huang CC, Ferrin TE. Tools for integrated sequence-structure analysis with UCSF Chimera. BMC Bioinformatics. 2006 Jul 12;7(1):339. Available from: http://bmcbioinformatics.biomedcentral.com/articles/10.1186/1471-2105-7-339

2. Maiti R, Van Domselaar GH, Zhang H, Wishart DS. SuperPose: a simple server for sophisticated structural superposition. Nucleic Acids Res. 2004 Jul 1;32(Web Server issue):W590-4. Available from: http://www.ncbi.nlm.nih.gov/pubmed/15215457

3. Johnson MS, Sutcliffe MJ, Blundell TL. Molecular anatomy: phyletic relationships derived from three-dimensional structures of proteins. J Mol Evol. 1990 Jan;30(1):43–59. Available from: http://www.ncbi.nlm.nih.gov/pubmed/2107323

4. Krissinel E, Henrick K, IUCr. Secondary-structure matching (SSM), a new tool for fast protein structure alignment in three dimensions. Acta Crystallogr Sect D Biol Crystallogr. 2004 Dec 1;60(12):2256–68. Available from: http://scripts.iucr.org/cgi-bin/paper?S0907444904026460
