# Supplementary material for: Electron cryo-microscopy of bacteriophage PR772 reveals the elusive vertex complex and the capsid architecture
Source: eLife. 2019 Sep 12;8:e48496. doi: 10.7554/eLife.48496 (PMC6750898; doi:10.7554/eLife.48496)

A symmetric reconstruction of the wild type PR772 by symmetry breaking using EMAN2. (**A**) Preliminary map generated by the asymmetric reconstruction of PR772. Note that all the vertices show the heteropentameric penton. (**B**) FSC curve for the reconstruction.


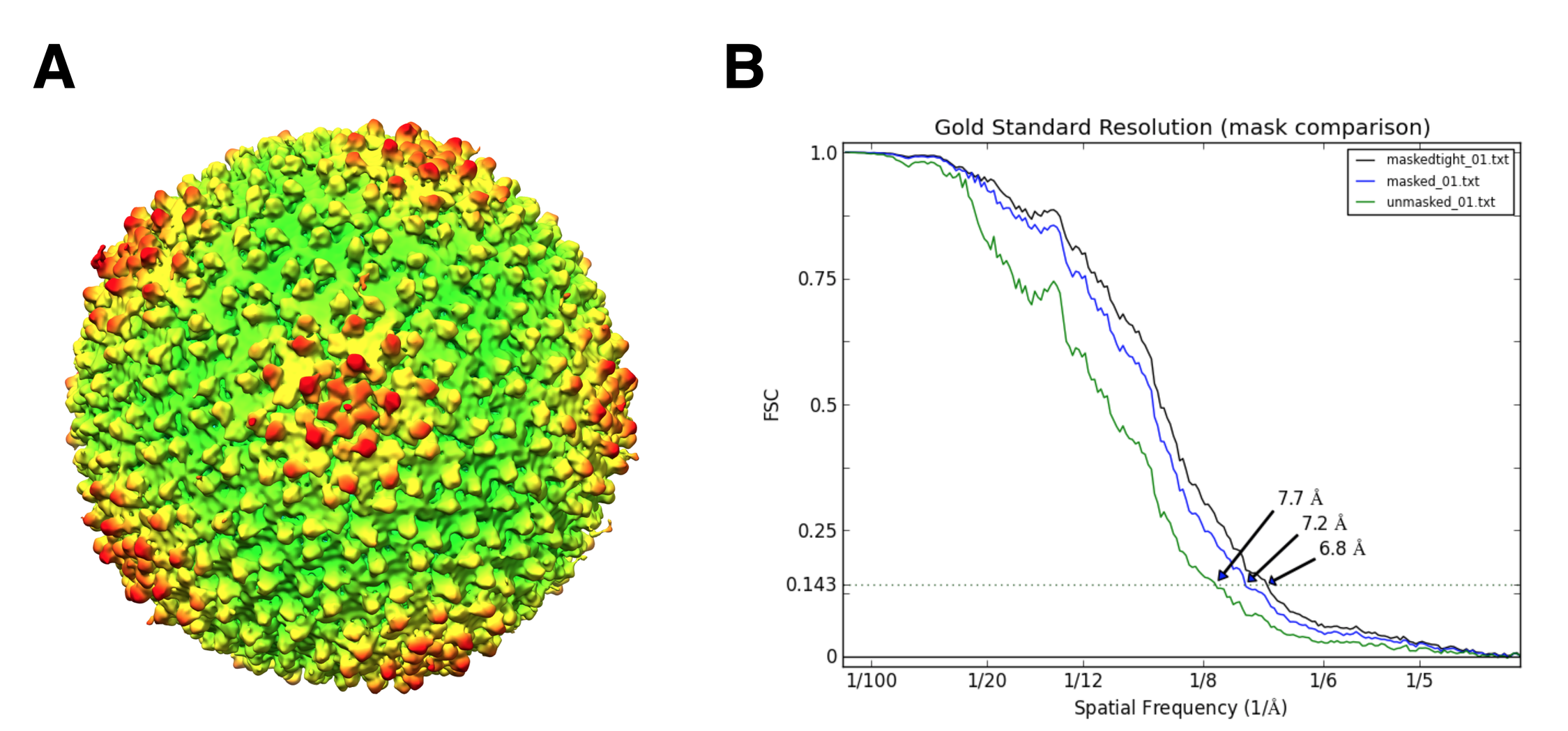

Supplement: Supplementary file 2. [file elife-48496-supp2.docx]
