# Supplementary material for: Electron cryo-microscopy of bacteriophage PR772 reveals the elusive vertex complex and the capsid architecture
Source: eLife. 2019 Sep 12;8:e48496. doi: 10.7554/eLife.48496 (PMC6750898; doi:10.7554/eLife.48496)

Flowchart of the 3D reconstruction of the icosehadrally averaged PR772 map. The preprocessed micrographs were used to auto-pick the particles using template matching. The auto-picked particles were extracted by 2x binning. The good particles were selected by inspecting the 2D class averages from the reference free 2D classification. An icosahedrally averaged ab-initio initial model was generated and used as a reference to do 3D classification. The particles from the dominant classes were selected and 3D refined. On reaching Nyquist, the particles from the 2x binned reconstruction were re-extracted without binning for further 3D refinement. The reference map was scaled accordingly.


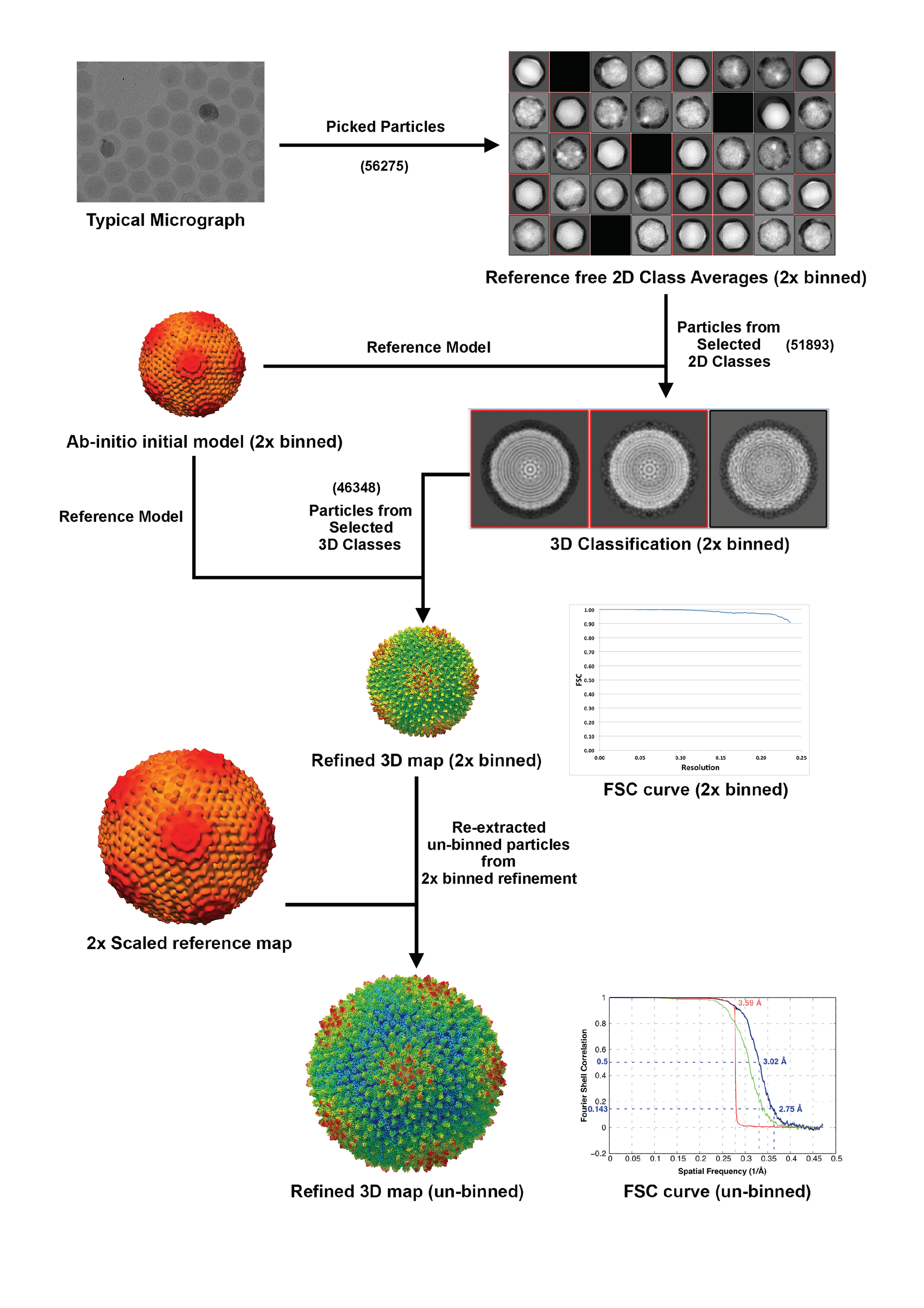

Supplement: Supplementary file 3. [file elife-48496-supp3.docx]
