# Supplementary material for: Electron cryo-microscopy of bacteriophage PR772 reveals the elusive vertex complex and the capsid architecture
Source: eLife. 2019 Sep 12;8:e48496. doi: 10.7554/eLife.48496 (PMC6750898; doi:10.7554/eLife.48496)
Supplement: Supplementary file 4. [file elife-48496-supp4.docx]

**Mtriage Summary**

| Map Resolution Estimates | Masked | Unmasked |
| --- | --- | --- |
| using map alone (d99) | 2.61 | 2.43 |
| using map alone (d9999) | 2.20 | 1.94 |
| using map alone (d99999) | 2.04 | 1.71 |
| comparing with model (d_model) | 2.90 | 3.00 |
| b_iso_overall | 40.00 | 30.00 |
| comparing with model (d_model_b0)  b_iso_overall=0 | 3.40 | 3.30 |
| d_fsc_model |  |  |
| FSC(map,model map)@0 | 2.25 | 2.37 |
| FSC(map,model map)@0.143 | 2.57 | 2.79 |
| FSC(map,model map)@0.5 | 2.89 | 49.04 |
| d99 (half map 1) | 3.51 | 2.76 |
| d99 (half map 2) | 3.51 | 2.76 |
| FSC(half map 1,2)@0.143 (d_fsc) | 2.71 | 2.70 |
|  |  |  |
| Radius used for mask smoothing: 2.43 | | |
| Supplied Resolution (based on [FSC@0.143](mailto:FSC@0.143) from Relion): 2.75 | | |

**Map-model CC (overall)**

| CC_mask | 0.8349 |
| --- | --- |
| CC_volume | 0.8348 |
| CC_main_chain | 0.8386 |
| CC_side_chain | 0.8085 |

Legend:

- **d_fsc** - highest resolution at which the experimental data are confident. Obtained from FSC curve calculated using two half-maps and taken at FSC=0.143.
- **d99** - resolution cutoff beyond which Fourier map coefficients are negligeably small. Calculated from the map.
- **d_model** - resolution cutoff at which the model map is the most similar to the target (experimental) map.
- **d_FSC_model** - resolution cutoff up to which the model and map Fourier coefficients are similar.
